# Zika in the United States of America and a Fateful 1969 Decision

**DOI:** 10.1371/journal.pntd.0004765

**Published:** 2016-05-26

**Authors:** Peter J. Hotez

**Affiliations:** 1 Sabin Vaccine Institute and Texas Children’s Hospital Center for Vaccine Development, National School of Tropical Medicine, Baylor College of Medicine, Houston, Texas, United States of America; 2 James A Baker III Institute for Public Policy, Rice University, Houston, Texas, United States of America; 3 Department of Biology, Baylor University, Waco, Texas, United States of America; University of California, Davis, UNITED STATES

## Abstract

The United States Gulf Coast’s current risk to Zika transmitted by *Aedes aegypti* mosquitoes can be traced back to some important federal health policy decisions made during the 1960s.

*Aedes aegypti*, the major mosquito responsible for the urban transmission of yellow fever, dengue, chikungunya, and now Zika virus infection, is believed to have originated in Africa, and likely was introduced into the New World in connection with the Atlantic slave trade from West Africa, probably beginning in the 1600s [1,2]. The New World’s first yellow fever outbreak was described in Barbados and the Yucatan Peninsula in 1647, while epidemics plagued the Southern US throughout the 18^th^ and 19^th^ centuries [2, 3].

Over the past five centuries yellow fever and dengue caused devastating and sometimes highly lethal outbreaks in the Western Hemisphere as far north as New York, until 1947 when the Pan American Sanitary Organization (the forerunner of the Pan American Health Organization of the World Health Organization) met in Buenos Aires to launch a comprehensive and coordinated campaign against *Ae aegypti* [4]. The PAHO Eradication Program relied on national programs of centralized organization and military-style campaigns that focused on source reduction to remove or empty containers with water where mosquitoes bred, and DDT spraying directed at mosquito breeding sites [4]. These efforts often required that health workers enter homes to conduct spraying and check for mosquito infestations [4]. The effectiveness depended on achieving high coverage rates through household access.

The results of the PAHO Eradication Program lasted almost two decades and produced impressive results. By 1962 the *Ae aegypti* mosquito was eradicated in almost 20 Latin American countries, including Brazil and all of the Central American countries [5], such that there were only 60 reported cases of dengue hemorrhagic fever reported between 1968 and 1980 [4]. Urban yellow fever rates similarly were dramatically reduced [6, 7].

Unfortunately those gains did not translate across the border into the United States. Despite the presence of *Ae aegypti* in all of the Gulf Coast states, and a history of repeated dengue fever epidemics in Texas the southeastern US during the 1920s, 30s, and 40s [2], throughout the years of the PAHO Eradication Program the US Government was mostly unresponsive to requests from Latin American and Caribbean (LAC) countries to participate in their campaign [5]. The fear among LAC countries was that reinfestation of the *Ae aegypti* mosquito from the US represented a constant threat to all of the gains made during the previous decades [5]. During this period it is believed that *Ae aegypti* was exported from the US into tropical regions of the Americas, in part due to the used tire trade [8].

Finally in 1965 the US federal government launched its *Ae aegypti* eradication program, which was administered through local and state health departments [5]. But the initiative was then dropped four years later due to lack of funds and political will [5]. Another reason cited beyond the costs was the low priority for the US Government given that the last yellow fever and dengue epidemics to occur in the continental US were in New Orleans, and happened sixty years previously (1905) and twenty years previously (1945), respectively [5]. Still another likely factor was logistical difficulties due to lack of access to private homes or cultural norms of privacy in the US.

Thus as shown in Fig 1 *Ae aegypti* was present throughout the Americas during the 1930s –from Argentina in the South extending as far north as the US Gulf Coast. However, by 1970 *Ae aegypti* had been mostly eradicated in the Americas except in the northernmost countries of tropical South America, some of the Caribbean islands, and the US Gulf Coast [2]. The US represented one of the last geographic reservoirs of *Ae aegypti* in the Americas!

**Fig 1:**
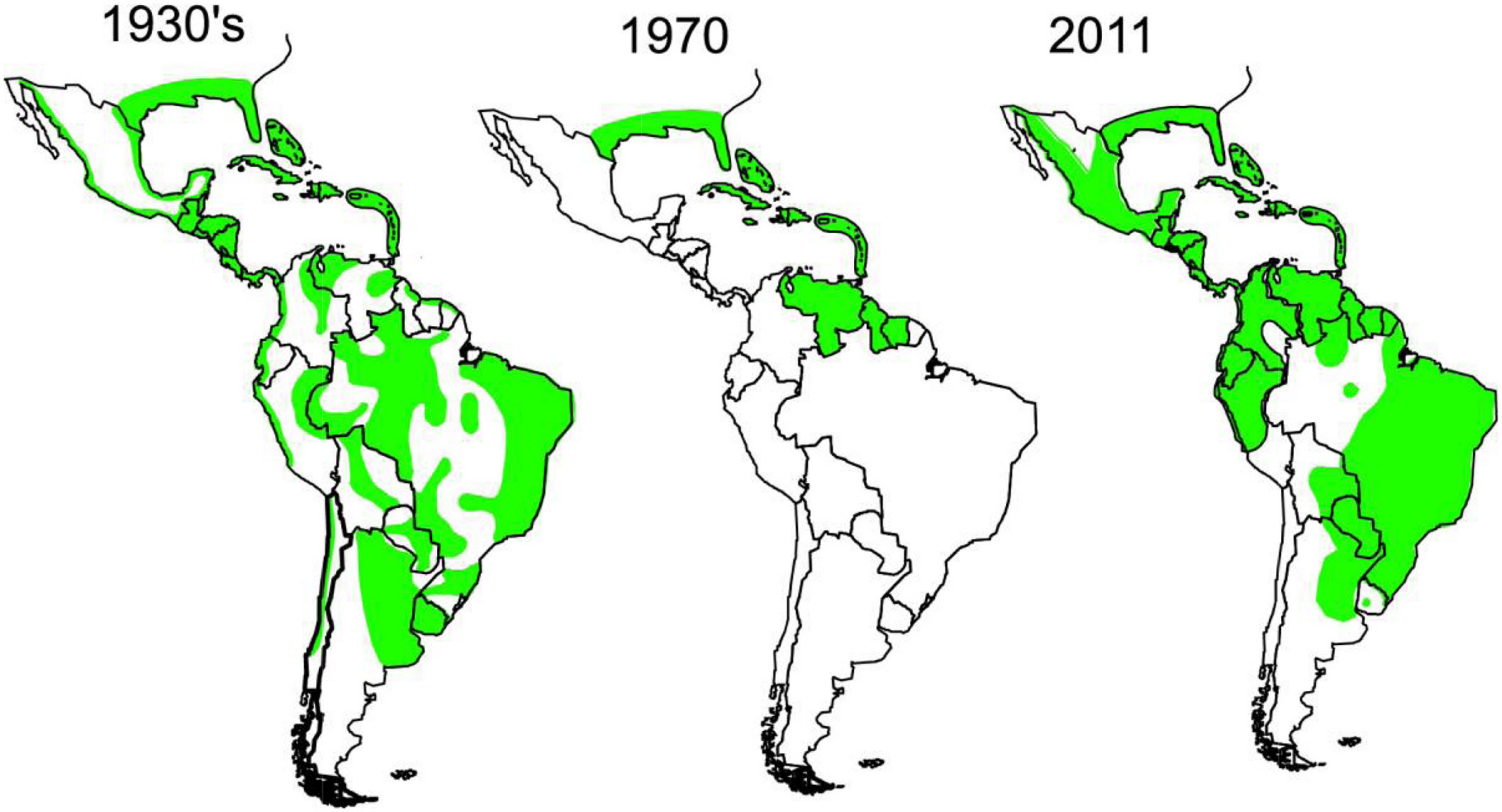
Distribution of *Aedes aegypti* in Western Hemisphere. From Gubler, Duane J. “Dengue, Urbanization and Globalization: The Unholy Trinity of the 21^st^ Century.” *Tropical Medicine and Health* 39.4 Suppl (2011): 3–11.*PMC*. Web. 4 Apr. 2016. http://www.ncbi.nlm.nih.gov/pmc/articles/PMC3317603/figure/F3/.

Subsequently, during the 1970s the political will to eradicate *Ae aegypti* also eroded in previously successful Latin American countries, resulting in mosquito densities increasing toward pre-campaign levels. Also promoting the return of *Ae aegypti* were key factors such as increased urbanization, rapid transportation, the rise of disposable and plastic products, and insecticide resistance. As a result, dengue re-emerged in Latin America and the Caribbean by the 1980s (after first arriving in epidemic form in Cuba in 1981) [2], followed by chikungunya virus infection in 2013 [9], and probably Zika virus infection in the same year [10].

Ultimately, the presence of *Ae aegypti* and associated arboviral diseases on the US Gulf Coast each represent a situation that appears to have remained unchanged for at least a century and probably much longer. There have also been ecological changes, as the introduction of the Asian tiger mosquito, *Aedes albopictus*, has displaced or co-habited with *Ae*. *aegypti* in some areas [11], but both vectors can transmit dengue, chikungunya, or Zika virus infections. In 2003, Houston, Texas experienced an outbreak of dengue [12]–the first time dengue struck a major US urban center in decades. It is an episode that could portend future risks from additional arbovirus infections entering the US. The areas of greatest risk include urban areas of the Gulf Coast, and also Hawaii and some parts of California [13].

Although *Ae aegypti* control is difficult and labor intensive and often involves house-to-house source reduction and spraying [14], the PAHO Eradication Program demonstrated its feasibility, provided adequate resources and commitment. The years between 1965 and 1969 presented a window of opportunity for the US to address its *Ae aegypti* problem and potentially establish a new generation of mosquito control expertise in America. Instead, much of the expertise in *Ae aegypti* control has become fragmented, or in some cases lost altogether. The recent emergence of Zika virus infection in Mesoamerica and the Caribbean may yet again force this important issue to the forefront of the US public health agenda. With adequate commitment of resources and defined goal, *Ae aegypti* control is doable and necessary [15, 16].

We can no longer remain complacent about the presence of *Ae aegypti* in the US. The current attention to Zika virus infection, the vulnerability of the US Gulf Coast, and the invasion of *Ae aegypti* into new areas like California demand our attention urgent commitment. While dengue and chikungunya are major neglected tropical disease threats unto themselves, the clear role of Zika virus infection in causing congenital microcephaly and fetal brain disruption sequence represents an unprecedented public health challenge in the US. The prospect of newborns with such birth defects on the Gulf Coast, California, or elsewhere on the continental US could create a public health crisis that might far outstrip the fear and panic linked to the three Ebola virus cases in Dallas, Texas that emerged in 2014. Accordingly, it may become necessary for local, state, and federal governments to embark on an unprecedented campaign against the *Ae aegypti* mosquito. While these activities might not closely resemble the Latin American programs of the 1960s, they will likely be more labor intensive and expensive than Culex mosquito control efforts as currently conducted, depending on local expertise and community cultural norms. It will be equally important for US public health authorities to maintain a vigilant global response to an ever-growing dengue pandemic possibly affecting hundreds of millions of people annually, and a new yellow fever outbreak in West Africa [17]. Indeed, arbovirus infections and other vector-borne diseases now represent some of the most important global health threats in this second decade of the 21^st^ century.
